# Supplementary material for: A Flexible Platform of Electrochemically Functionalized Carbon Nanotubes for NADH Sensors
Source: Sensors (Basel). 2019 Jan 26;19(3):518. doi: 10.3390/s19030518 (PMC6386930; doi:10.3390/s19030518)
Supplement: Supplementary file 1 [file sensors-19-00518-s001.pdf]

Flexible platform of electrochemically functionalized carbon nanotubes for NADH sensors

Aranzazu Heras[1,*], Fabio Vulcano[2,3], Jesuz Garoz-Ruiz[1], Nicola Porcelli[2], Fabio Terzi[2], Alvaro Colina[1], Renato Seeber [2,3], and Chiara Zanardi[2,3,*]

1 Department of Chemistry, Universidad de Burgos, Pza. Misael Baneuelos s/n, E-09001 Burgos, Spain
2 Department of Chemical and Geological Sciences, Università di Modena e Reggio Emilia, Via G. Campi 103, 41125 Modena, Italy; fabio.vulcano@unimore.it, fabio.terzi@unimore.it, renato.seeber@unimore.it
3 Institute of Organic Synthesis and Photoreactivity (ISOF), National Research Council of Italy (CNR), via P. Gobetti 101 - 40129 Bologna, Italy.
* Correspondence: chiara.zanardi@unimore.it, maheras@ubu.es

SUPPORTING INFORMATION

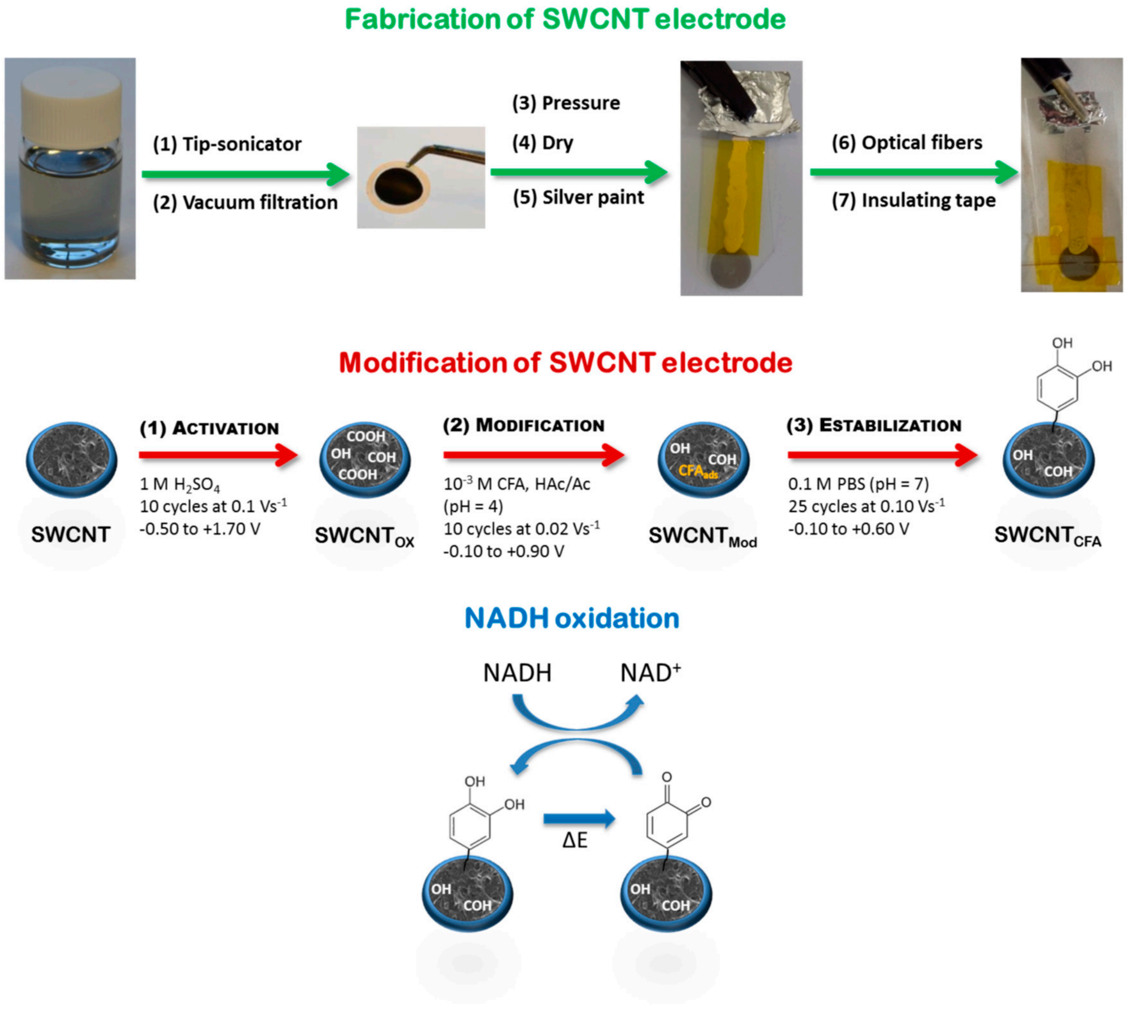

Figure S1. Schematic representation of the different steps involved in electrode fabrication (top), functionalization of SWCNTs (middle) and NADH oxidation (bottom).

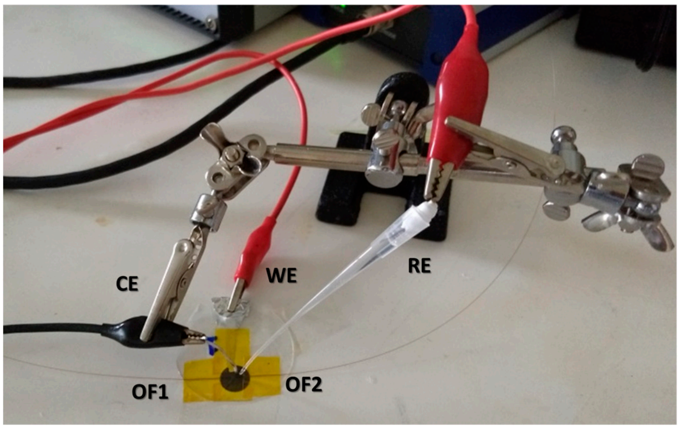

Figure S2. Photograph of the UV/Vis absorption spectroelectrochemical set-up in parallel configuration. WE: SWCNT working electrode, CE: Pt counter electrode, RE: Ag/AgCl/KCl 3M reference electrode, OF1: naked optical fibre that guides the light beam from the source cell to the solution, OF2: naked optical fiber that guides the light beam from the solution to the spectrometer.

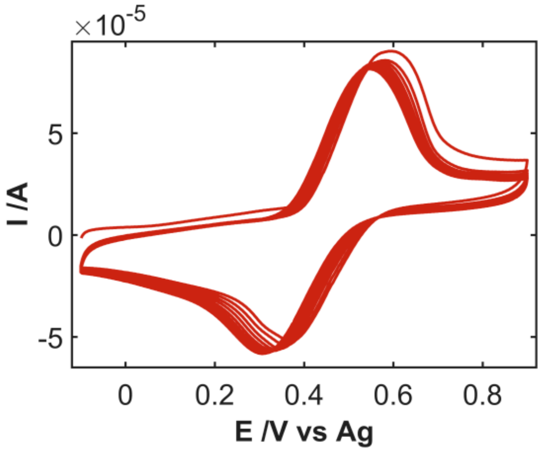

Figure S3. CV response of 10^{-3} M CFA, 0.1 M acetic acid solution at $SWCNT_{ox}$ electrode between -0.10 and +0.90 V at 0.02 Vs^{-1}.

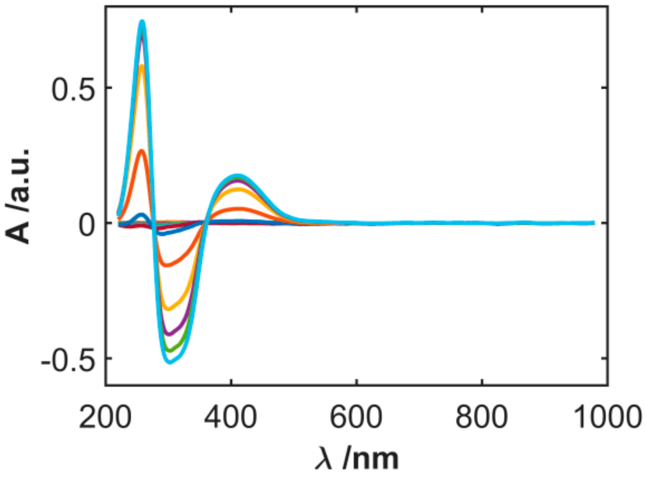

Figure S4. UV-Vis absorption spectra recorded during the first scan toward positive potential values at $SWCNT_{ox}$ electrode in 10^{-3} M CFA, 0.1 M acetic acid solution, between -0.10 and +0.90 V at 0.02 Vs^{-1}.

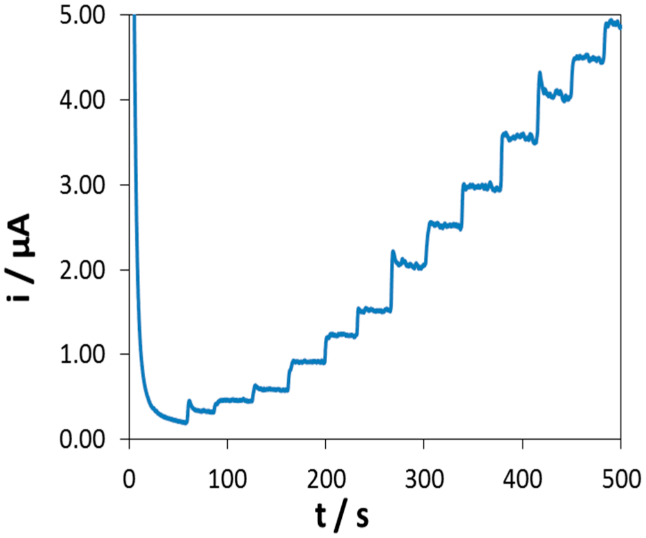

Figure S5. Amperometric response recorded with $SWCNT_{CFA}$ at +0.30 V in stirred 0.1 M PBS, by subsequent additions of defined aliquots of a NADH solution.

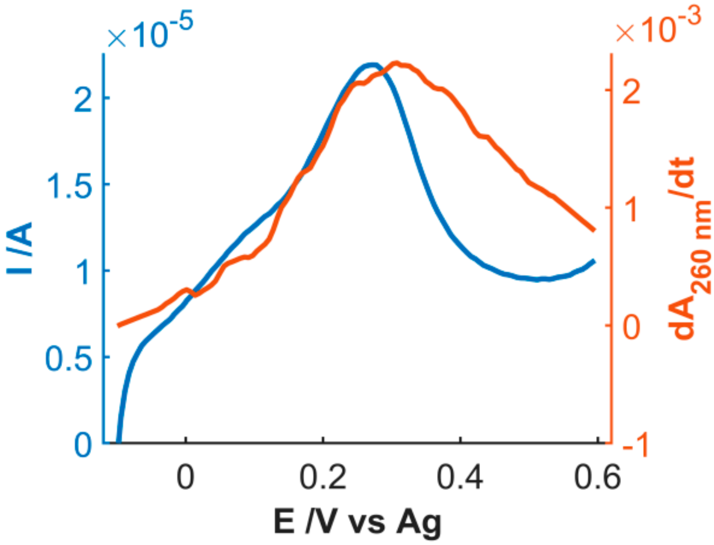

Figure S6. Comparison of LSV and derivative voltabsorptogram at 260 nm during the oxidation of $3 \cdot 10^{-4}$ M NADH, 0.1 M PBS.

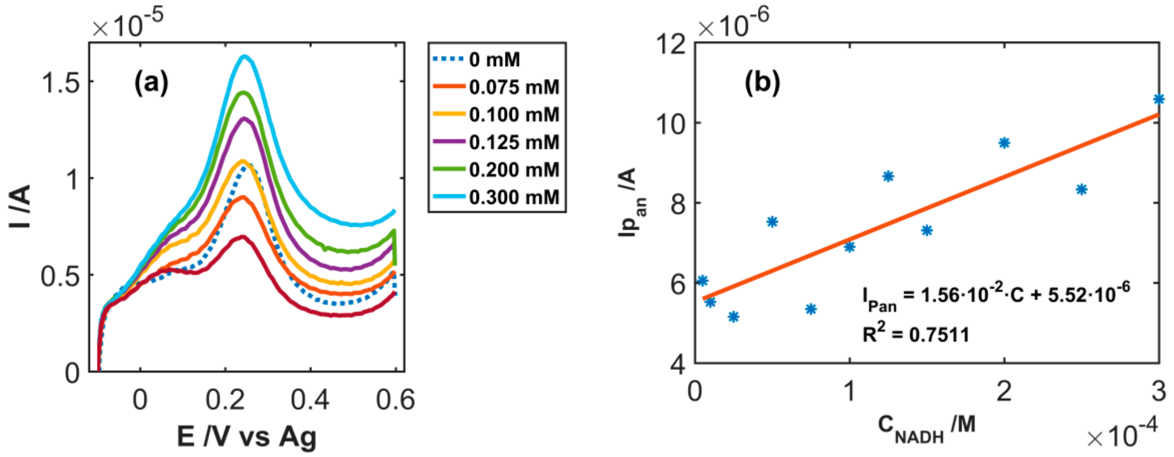

Figure S7. (a) LSV registered during the oxidation of $2 \cdot 10^{-4}$ M NADH in 0.1 M PBS. (b) Calibration curve of the current peak values *vs* the NADH concentration.
